# A classification modeling approach for determining metabolite signatures in osteoarthritis

**DOI:** 10.1371/journal.pone.0199618

**Published:** 2018-06-29

**Authors:** Jason S. Rockel, Weidong Zhang, Konstantin Shestopaloff, Sergei Likhodii, Guang Sun, Andrew Furey, Edward Randell, Kala Sundararajan, Rajiv Gandhi, Guangju Zhai, Mohit Kapoor

**Affiliations:** 1 Arthritis Program, Toronto Western Hospital, University Health Network, University of Toronto, Toronto, Ontario, Canada; 2 Krembil Research Institute, University Health Network, Toronto, Ontario, Canada; 3 Discipline of Genetics, Faculty of Medicine, Memorial University of Newfoundland, St John’s, Newfoundland, Canada; 4 School of Pharmaceutical Sciences, Jilin University, Changchun, P.R. China; 5 Department of Laboratory Medicine, Faculty of Medicine, Memorial University of Newfoundland, St John’s, Newfoundland, Canada; 6 Discipline of Medicine, Faculty of Medicine, Memorial University of Newfoundland, St John’s, Newfoundland, Canada; 7 Department of Surgery, Faculty of Medicine, Memorial University of Newfoundland, St John’s, Newfoundland, Canada; 8 Department of Surgery, University of Toronto, Toronto, Ontario, Canada; 9 Menzies Research Institute, University of Tasmania, Hobart, Tasmania, Australia; 10 Department of Laboratory Medicine and Pathobiology, University of Toronto, Ontario, Canada; University of Umeå, SWEDEN

## Abstract

Multiple factors can help predict knee osteoarthritis (OA) patients from healthy individuals, including age, sex, and BMI, and possibly metabolite levels. Using plasma from individuals with primary OA undergoing total knee replacement and healthy volunteers, we measured lysophosphatidylcholine (lysoPC) and phosphatidylcholine (PC) analogues by metabolomics. Populations were stratified on demographic factors and lysoPC and PC analogue signatures were determined by univariate receiver-operator curve (AUC) analysis. Using signatures, multivariate classification modeling was performed using various algorithms to select the most consistent method as measured by AUC differences between resampled training and test sets. Lists of metabolites indicative of OA [AUC > 0.5] were identified for each stratum. The signature from males age > 50 years old encompassed the majority of identified metabolites, suggesting lysoPCs and PCs are dominant indicators of OA in older males. Principal component regression with logistic regression was the most consistent multivariate classification algorithm tested. Using this algorithm, classification of older males had fair power to classify OA patients from healthy individuals. Thus, individual levels of lysoPC and PC analogues may be indicative of individuals with OA in older populations, particularly males. Our metabolite signature modeling method is likely to increase classification power in validation cohorts.

## Introduction

Commencing at age 50, there is a steep increase in the incidence of symptomatic osteoarthritis (OA) and the number of individuals undergoing total knee replacement (TKR) [1–3]. Individuals of high body mass index (BMI) and female sex also have increased risk of TKR due to primary OA [4, 5]. The World Health Organization defines individuals with BMI ≥ 30 as obese. There are currently no effective biomarkers to identify individuals with advanced OA.

The metabolome represents the cumulative output of metabolic processes occurring within an individual and includes compounds such as lipids and amino acids, among others. In addition to the amino acid arginine [6], select lysophosphatidylcholine (lysoPC) to phosphatidylcholine (PC) analogue ratios are altered in plasma from patients with OA compared to healthy adult volunteers (HV) and the ratio of total lysoPCs to PCs are predictive of TKR in 10 years follow-up [7]. It is unclear, however if a signature of individual metabolite levels, specifically of lysoPC or PC analogues, in a combined signature, is capable of classifying OA from healthy individuals. In addition, optimal methods to help improve predictive metabolite selection and prediction modeling in cross-sectional cohorts to aid in successful prediction in external validation cohorts have not been investigated.

In this study, we sought to determine if a signature of metabolite levels could be predictive of OA vs healthy volunteers using a selection and modeling method to improve selection and predictive consistency. Using plasma from a cohort of patients undergoing TKR surgery due to primary OA and HV, we measured lysoPC and PC analogues. Stratifying along age, sex and BMI, we determined unique signatures based on each stratum using systematic univariate modeling followed by prediction analysis using various multivariate modeling algorithms for comparison. Thus, we present a method to investigate demographically-stratified populations from a single cross-sectional cohort to determine the best possible predictive metabolite signature of individual metabolite levels that can be used in future validation studies.

## Materials and methods

### Study participants

Patients receiving TKR due to primary OA were obtained from the Newfoundland Osteoarthritis Study (NFOAS) [8]. HV were from the The Complex Diseases in the Newfoundland population: Environment and Genetics (CODING) study [9]. Both OA patients and HV were from Newfoundland & Labrador, Canada. Knee OA diagnosis was made based on American College of Rheumatology clinical criteria for classification of idiopathic OA of the knee [10] and judgment of attending orthopedic surgeons. Controls were those without an OA diagnosis in any joints based on medical information collected by a self-administered questionnaire. The distribution of clinical and demographic variables in strata and a comparison between OA and HV individuals is shown in Table 1.

**Table 1:** Demographics of the Newfoundland cohort stratified groups consisting of healthy volunteers (HV) and patients undergoing total knee replacement for osteoarthritis (OA).

Stratum	Total (n)	OA (n)	HV (n)	Females (n)	Females OA (n)	Females HV (n)	P-value Males:Females OA vs. HV	Age ± SD	Age OA ± SD	Age HV ± SD	P-value Age OA vs. HV	BMI ± SD	BMI OA ± SD	BMI HV ± SD	P-value BMI OA vs. HV
All	346	152	194	187	77	110	0.312	56.1 ± 12.8	63.8 ± 7.5	50.0 ± 12.8	< 0.001	30.0 ± 5.4	31.8 ± 5.6	28.6 ± 4.9	< 0.001
Males	159	75	84					57.3 ± 12.1	63.6 ± 7.9	51.7 ± 12.4	< 0.001	29.7 ± 4.7	31.1 ± 4.9	28.5 ± 4.2	< 0.001
Females	187	77	110					55.0 ± 13.3	64.1 ± 7.1	48.7 ± 12.9	< 0.001	30.3 ± 6.0	32.5 ± 6.1	28.7 ± 5.4	< 0.001
BMI ≥ 30	172	89	83	98	47	51	0.323	56.0 ± 12.1	62.9 ± 5.9	48.5 ± 12.7	< 0.001	34.0 ± 4.2	35.0 ± 5.0	33.0 ± 2.7	0.002
BMI < 30	174	63	111	89	30	59	0.586	56.2 ± 13.4	65.2 ± 9.1	51.1 ± 12.8	< 0.001	26.0 ± 3.2	27.4 ± 2.4	25.3 ± 3.3	< 0.001
Age > 50	250	148	102	128	76	52	1.000	62.5 ± 6.9	64.4 ± 6.7	59.9 ± 6.2	< 0.001	30.7 ± 5.2	32.0 ± 5.5	28.8 ± 3.8	< 0.001
Males Age > 50	122	72	50					62.6 ± 7.0	64.5 ± 6.5	59.8 ± 6.8	< 0.001	30.3 ± 4.4	31.2 ± 4.9	29.1 ± 3.0	0.005
Females Age > 50	128	76	52					62.5 ± 6.7	64.3 ± 6.9	59.9 ± 5.6	< 0.001	31.0 ± 5.8	32.7 ± 6.0	28.5 ± 4.5	< 0.001
Age > 50, BMI ≥ 30	131	89	42	70	47	23	0.983	61.5 ± 6.5	62.9 ± 5.9	58.6 ± 6.7	0.001	34.1 ± 4.4	35.0 ± 5.0	32.2 ± 2.1	< 0.001
Age > 50, BMI < 30	119	59	60	58	29	29	1.000	63.7 ± 7.1	66.6 ± 7.2	60.8 ± 5.7	< 0.001	26.9 ± 2.6	27.4 ± 2.3	26.4 ± 2.8	0.031

Ratio of males to females, age or BMI in the OA vs. HV groups within each stratified group was determined by chi-square tests. P-values < 0.05 are considered significant. Number of individuals (n); age (in years); body mass index (BMI; in kg/m^2^); SD, standard deviation.

### Metabolite profiling, signature determination, and predictive modeling

Blood samples were collected after minimum 8 hours of fasting. Blood was collected into K_2_EDTA-plasma tubes. EDTA-plasma was separated from whole blood by centrifugation at 1500 rcf for 10 mins at 4°C. Plasma was aliquoted at stored at -80°C until use. Plasma was thawed on ice and metabolite profiling was performed using Liquid Chromatography(LC)/Mass Spectrometry (MS)/MS using the Waters XEVO TQ MS Ultra Performance LC/MS/MS system (Waters Limited, Mississauga, Ontario, Canada) coupled with Biocrates AbsoluteIDQ p180 kit. All analytical metabolite quantification was performed using the Absolute IDQ-coupled MetIDQ software package (Biocrates Life Sciences AG, Austria), as described [8]. Only lysoPC and PC analogue concentrations were used for analysis in this study.

Metabolite concentrations were adjusted for batch effects and log (x+1) transformed to normalize variable distributions for modeling using linear effects. The cohort was stratified based on age (≤ 50 or > 50 years), BMI (≥ 30 or < 30 kg/m^2^), and/or sex. The information for each stratum is presented in Table 1.

Subpopulations (OA vs. HV, stratified by age) were assessed for metabolite variance homogeneity to check for structural differences. Homogeneity was tested using PERMDISP2 [11] and significance was measured using Tukey’s HSD test. For model building, we pre-selected individual metabolites within each of the stratified populations using predictive area under the curve (AUC) in a logistic regression using a non-parametric bootstrap [12]. We randomly sampled N individuals with replacement to generate 1000 training sets to which we fit a logistic regression with single metabolites, and then estimated predictive AUCs on individuals not included in the corresponding training set. Under the bootstrap, this is about 1/3 of the samples, constituting the test set. Using the replicates, we generated an empirical distribution of AUCs for each metabolite and selected those with consistent predictive power (AUC > 0.5 at the 2.5% quantile).

**Fig 1:**
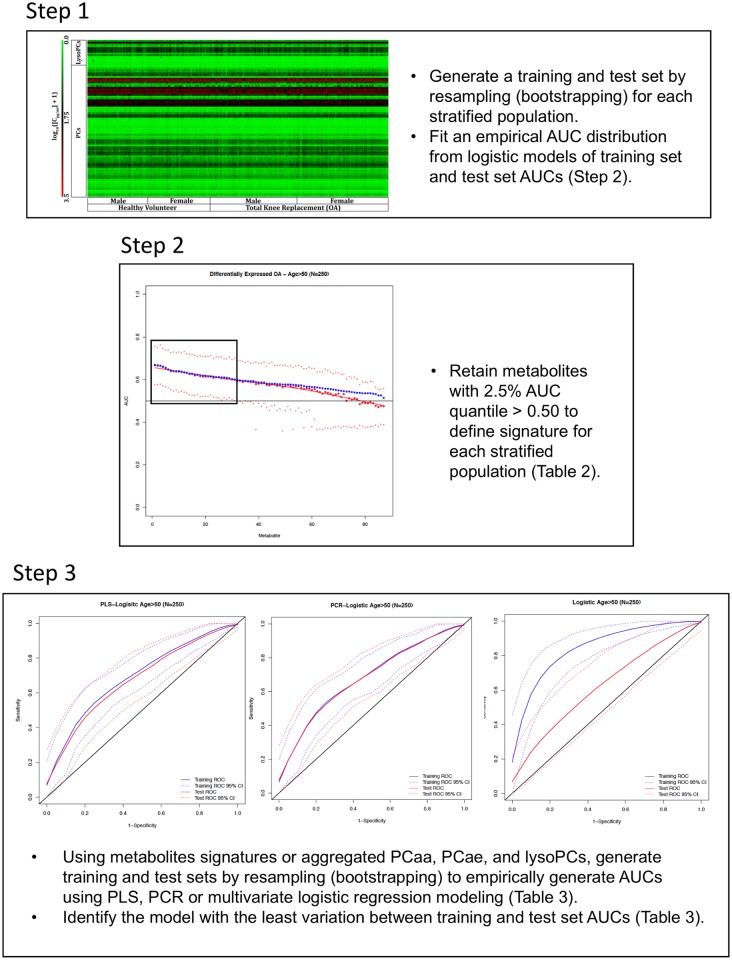
A stepwise approach to metabolite signature identification and predictive model optimization using stratified populations from a single cohort. The following stepwise approach includes data from the age > 50 years stratified population and is representative of results generated for each subpopulation. AUC, area under the curve; lysophosphatidylcholine (lysoPC); diacyl-phosphatidylcholine (PCaa); acyl-alkylphosphatidylcholine (PCae); partial least squares with logistic regression (PLS); principal component analysis with logistic regression (PCR).

Metabolites above the cutoff for each stratified population were used as inputs into three predictive algorithms for classification: principal component regression with logistic regression (PCR), partial least squares regression (PLS) with logistic regression, and simple logistic regression. PLS and PCR approaches were used for dimensionality reduction as metabolites tended to be correlated. The use of multiple metabolites projected onto components of correlation for PLS and variation for PCR is to achieve a more robust signal, compared to reducing metabolite lists to achieve a higher AUC, which may be specific to the dataset and thus overfit the model. For each modeling algorithm, the bootstrap process was repeated 1000 times to generate an empirical distribution of AUCs. For PCR and PLS, the number of components was selected by minimizing overfit, as measured by the differences between average training and test set AUCs from the bootstrap. The results indicated that the first principal component was optimal in both models. Aggregate concentrations of metabolite groups [(lysoPC, diacyl PC (PCaa), acyl-alkyl PC (PCae)] from all measured metabolites were also used in separate logistic models. The process was repeated for all strata. The modeling process is presented in Fig 1. We have also provided the statistical code used for analysis using R package (https://www.r-project.org; S1 Document).

### Study Approval

The study was approved by the Health Research Ethics Authority (HREA) of Newfoundland & Labrador (reference number **11.311**) and written informed consent was obtained from all participants prior to inclusion in the study.

## Results

The cohort utilized for the study is presented in Table 1. A total of 346 individuals (152 OA, 194 HV; 159 males, 187 females) were studied. The OA population was older and had higher BMI (all P < 0.05), but the groups had similar sex distributions. Since age was significantly different, our study focused on individuals over 50 years old. This reduced the mean difference in age between OA and HV to 4.5 years compared to 13.6 years in the entire cohort (Table 1). Furthermore, variance of metabolite levels between age-stratified cohorts was significantly different (P < 0.05) whereas the variance of metabolite levels between OA and HV individuals over age 50 was not significantly different (P > 0.05), further suggesting a need for age stratification. The variance of metabolite levels between OA and HV individuals in BMI-stratified groups, either in the entire cohort or in individuals over the age of 50, was not significantly different (P > 0.05). This suggested that age, rather than BMI was the major confounder, even though BMI between HV and OA individuals was significantly different in stratification of age > 50 years with BMI ≥ 30 or BMI < 30 (Table 1).

**Fig 2:**
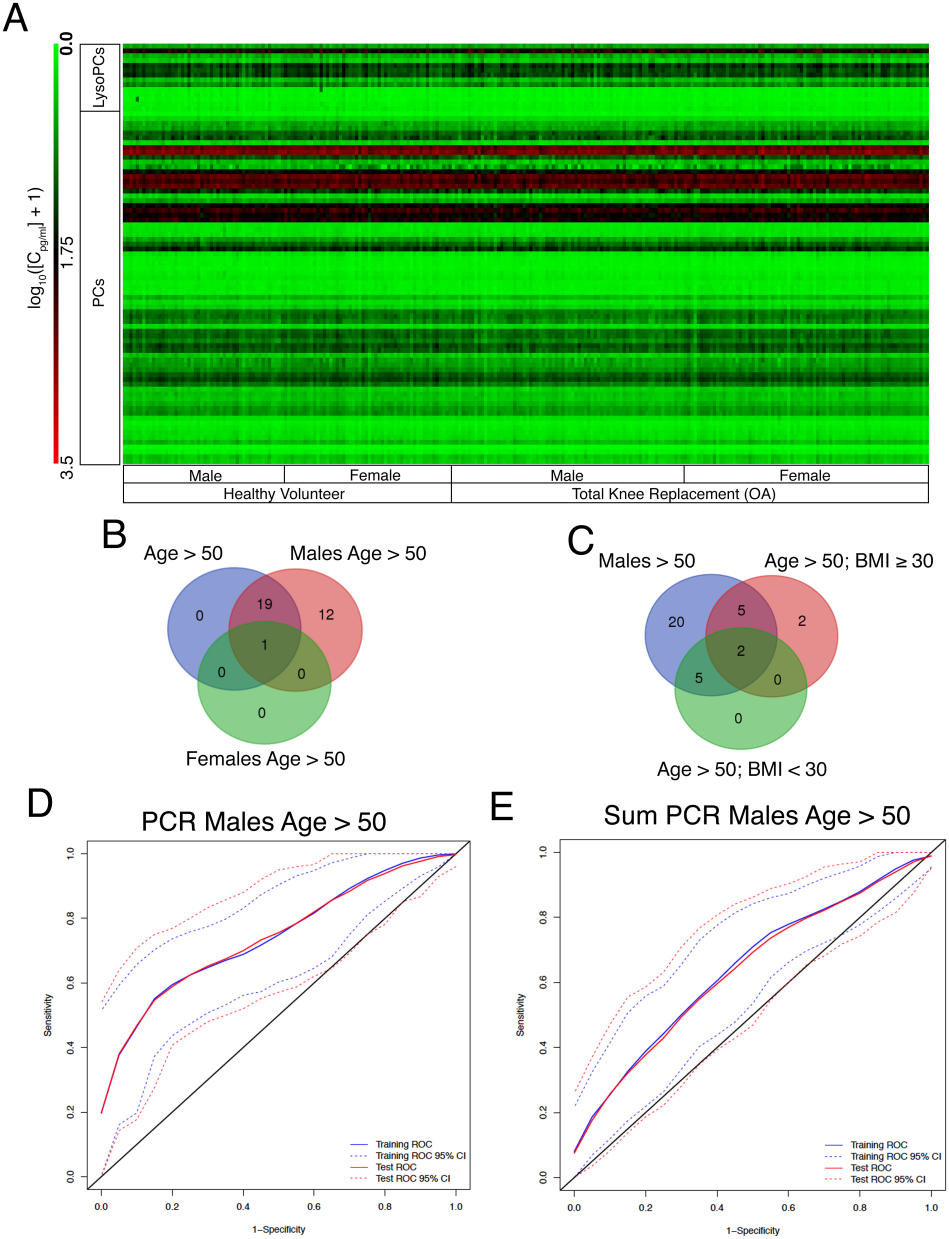
A discrete lysoPC and PC signature of metabolites from males over the age of 50 was dominant in individuals over the age of 50 years and was indicative of males with OA versus HV. (A) Heat-map of the stratified cohort of individuals overs the age of 50 years separated by sex and total knee replacement due to osteoarthritis (OA) vs healthy adult volunteers (HV). (B & C) Venn diagrams generated by metabolite signatures (Table 2) from males, females and all individuals over the age of 50 years (B) or males, individuals with body mass index (BMI) ≥ 30 or BMI < 30 kg/m^2^ (C). (D & E) AUC curves generated by principal component with logistic regression (PCR) modeling using the metabolite signature (D) or aggregate sum of lysophosphatidylcholine (lysoPC), diacyl-phosphatidylcholine (PCaa) and acyl-alkylphosphatidylcholine (PCae) analogues (E) from the male age > 50 years stratified population. Blue lines represent training set area under the curve (AUC). Red lines represent test set AUC. Dotted lines are 95% confidence intervals.

Qualitatively, we were unable to identify overt trends between males, females, HV and OA patients over 50 years old using heat-map analysis (Fig 2A), suggesting that smaller changes in individual metabolite levels are important for detecting OA. We therefore analyzed each stratum population to identify individual metabolites predictive of OA using univariate receiver operator AUC analysis (Table 2). A list of predictive metabolites for OA was identified using bootstrapped logistic regression analysis (Fig 1). Most of the strata generated unique signatures of OA-indicative metabolites, except for females and individuals with BMI < 30 kg/m^2^, which did not have any metabolites that met our selection criteria (Table 2).

**Table 2:** Metabolites with a 2.5% quantile area under the receiver-operator curve ≥ 0.5 determined by bootstrapped logistic regression of the stratified study population described in Table 1.

Metabolite	All	Age > 50	Males	Age > 50 Males	Females	Age > 50 Females	BMI ≥ 30	Age > 50 BMI ≥ 30	BMI < 30	Age > 50 BMI < 30
lysoPCaC16:0								0.66		
lysoPCaC28:1	0.6	0.64	0.64	0.67						0.66
PCaaC28:1			0.62	0.65						
PCaaC32:3	0.61	0.67	0.69	0.70		0.66	0.62	0.69		0.64
PCaaC34:3		0.62	0.65	0.68						
PCaaC36:0	0.6	0.62	0.63	0.66						
PCaaC36:2				0.65						
PCaaC36:5		0.61		0.69						
PCaaC36:6	0.59	0.64	0.64	0.70						
PCaaC38:0	0.59	0.64	0.67	0.72						
PCaaC38:5		0.61	0.64	0.68						
PCaaC38:6		0.61		0.65						
PCaaC40:1		0.61	0.63	0.67						
PCaaC40:2				0.65						
PCaaC40:6				0.65						
PCaaC42:0			0.62							
PCaaC42:2	0.59	0.63		0.66						0.65
PCaaC42:5				0.66						
PCaeC30:1			0.64	0.68						
PCaeC30:2	0.59	0.63	0.65	0.68						
PCaeC32:2	0.6	0.65	0.65	0.69				0.68		
PCaeC34:0		0.6								
PCaeC34:1		0.61								
PCaeC34:2		0.62		0.65						
PCaeC34:3		0.62		0.64						
PCaeC36:2	0.59	0.64	0.62	0.67				0.66		
PCaeC36:3	0.59	0.64	0.63	0.66				0.67		
PCaeC38:0	0.61	0.66	0.68	0.74						0.67
PCaeC38:1		0.61		0.64						
PCaeC38:2	0.6	0.66	0.65	0.69						0.67
PCaeC38:3		0.61						0.65		
PCaeC38:5		0.6								
PCaeC38:6	0.6	0.65	0.66	0.72				0.64		
PCaeC40:1		0.63		0.65						0.67
PCaeC40:2		0.61		0.67						
PCaeC40:5	0.61	0.63	0.63	0.67				0.67		
PCaeC40:6	0.62	0.67	0.64	0.7				0.67		0.63
PCaeC42:2		0.61								
PCaeC42:3		0.61		0.64						

Age (in years), body mass index (BMI; in kg/m^2^), lysophosphatidylcholine (lysoPC), diacyl PC (PCaa), acyl-alkyl PC (PCae).

Comparing signatures obtained from stratum age > 50 years old to the same aged populations also stratified based on sex, we determined that the signature identified in males age > 50 years old encompassed all of the metabolites identified within the age > 50 years population, along with the single metabolite identified for the age > 50 years female population (Fig 2B, Table 2). The signature from males age > 50 years old stratum also encompassed all but 2 metabolites derived from the age > 50 years with BMI ≥ 30 or < 30 kg/m^2^ stratified populations (Fig 2C). This suggests that lysoPC and PC analogues have most power to detect OA in older males.

Next, we investigated multivariate algorithms to identify ones with the most consistent classification power (Fig 1, Table 3). Analyzing differences in AUC we found that multivariate logistic modeling was poor at maintaining predictive consistency. For principal component with logistic regression (PCR) and partial least squares with logistic regression (PLS) approaches on the top component, PCR was slightly more consistent than PLS modeling based on absolute mean differences, being more consistent at the 2.5% and 50% quantiles, but slightly worse at the 97.5% quantile. PCR modeling also tended to be more conservative, as training set AUCs were marginally smaller compared to PLS (Table 3). Based on PCR modeling, we found that the males > 50 years old signature was most accurate in differentiating OA and HV, with a median AUC of 0.751 and 0.752, for the training and test sets respectively (Fig 2D, Table 3).

**Table 3:** Model area under the receiver-operator curve values (AUC) of the 2.5%, 50% and 97.5% quantiles generated from bootstrapped multivariate analysis of metabolites determined to be predictive from univariate analysis of stratified groups of study participants described in Table 1.

Model	All Train	All Test	All Difference	Age > 50 Train	Age > 50 Test	Age > 50 Difference	Males Train	Males Test	Males Difference	Males Age > 50 Train	Males Age > 50 Test	Males Age > 50 Difference	Age > 50, BMI ≥ 30 Train	Age > 50, BMI ≥ 30 Test	Age > 50, BMI ≥ 30 Difference	Age > 50, BMI < 30 Train	Age > 50, BMI < 30 Test	Age > 50, BMI < 30 Difference	Mean Absolute Difference (quantile)	Mean Absolute Difference (all)
pls 2.5%	0.595	0.563	0.032	0.630	0.589	0.041	0.647	0.588	0.059	0.684	0.625	0.059	0.645	0.573	0.072	0.617	0.562	0.055	0.053	
pls 50%	0.649	0.641	0.008	0.693	0.678	0.015	0.724	0.703	0.021	0.768	0.751	0.017	0.739	0.710	0.029	0.705	0.689	0.016	0.018	0.028
pls 97.5%	0.709	0.715	-0.006	0.753	0.762	-0.009	0.791	0.812	-0.021	0.844	0.859	-0.015	0.827	0.836	-0.009	0.786	0.810	-0.024	0.014	
																				
pcr 2.5%	0.586	0.566	0.020	0.613	0.592	0.020	0.630	0.589	0.041	0.659	0.623	0.036	0.560	0.545	0.015	0.597	0.564	0.032	0.027	
pcr 50%	0.643	0.645	-0.002	0.680	0.679	0.001	0.711	0.709	0.002	0.751	0.752	-0.001	0.707	0.701	0.007	0.696	0.691	0.005	0.003	0.018
pcr 97.5%	0.706	0.721	-0.015	0.745	0.763	-0.018	0.784	0.816	-0.032	0.835	0.865	-0.029	0.810	0.831	-0.020	0.780	0.817	-0.037	0.025	
																				
log 2.5%	0.665	0.512	0.153	0.799	0.528	0.271	0.758	0.472	0.286	0.830	0.412	0.418	0.700	0.481	0.219	0.666	0.476	0.190	0.256	
log 50%	0.717	0.601	0.116	0.852	0.627	0.225	0.830	0.599	0.231	0.972	0.587	0.386	0.794	0.639	0.155	0.751	0.622	0.129	0.207	0.203
log 97.5%	0.771	0.676	0.096	0.897	0.720	0.176	0.892	0.728	0.164	1.000	0.741	0.259	0.872	0.779	0.094	0.833	0.754	0.079	0.145	
																				
sum pls 2.5%	0.538	0.501	0.038	0.572	0.539	0.033	0.580	0.527	0.052	0.623	0.556	0.068	0.583	0.468	0.115	0.539	0.474	0.065	0.062	
sum pls 50%	0.588	0.581	0.007	0.638	0.628	0.011	0.657	0.641	0.015	0.704	0.692	0.012	0.661	0.627	0.033	0.621	0.611	0.010	0.015	0.033
sum pls 97.5%	0.644	0.662	-0.018	0.700	0.725	-0.025	0.733	0.755	-0.021	0.780	0.809	-0.029	0.739	0.769	-0.030	0.721	0.735	-0.013	0.023	
																				
sum pcr 2.5%	0.514	0.498	0.016	0.553	0.536	0.017	0.557	0.527	0.030	0.598	0.553	0.044	0.514	0.432	0.082	0.521	0.480	0.042	0.039	
sum pcr 50%	0.577	0.578	-0.001	0.630	0.625	0.005	0.648	0.640	0.008	0.691	0.689	0.003	0.611	0.604	0.008	0.616	0.615	0.000	0.004	0.023
sum pcr 97.5%	0.639	0.662	-0.023	0.695	0.723	-0.028	0.727	0.756	-0.029	0.777	0.802	-0.024	0.709	0.738	-0.030	0.719	0.745	-0.026	0.027	
																				
sum log 2.5%	0.559	0.504	0.055	0.600	0.538	0.063	0.599	0.515	0.084	0.647	0.549	0.098	0.613	0.517	0.096	0.562	0.432	0.130	0.088	
sum log 50%	0.615	0.586	0.029	0.667	0.632	0.035	0.679	0.631	0.047	0.735	0.686	0.050	0.708	0.654	0.054	0.649	0.569	0.080	0.049	0.051
sum log 97.5%	0.672	0.660	0.012	0.730	0.722	0.008	0.758	0.737	0.021	0.817	0.803	0.014	0.797	0.790	0.007	0.737	0.702	0.035	0.016	

AUC values were generated using partial least squares (pls), principal component analysis and logistic regression (pcr) and multivariate logistic regression (log) alone. Aggregated lysphosphatidylcholine, diacyl-phosphatidylcholine (PCaa) and acyl-alkylphosphatidylcholine (PCae) concentrations were also modelled (sum) in the same manner for each stratified group. Differences in training and test set were calculated and the mean absolute difference across all stratified groups was calculated for each quantile and for all quantiles to identify the model with least amount of overfitting between bootstrapped test and training sets. Age (in years), body mass index (BMI; in kg/m^2^).

Finally, we evaluated whether all measured phospholipids aggregated by type, namely lysoPCs, PCaas, and PCaes, could be used as predictors of OA (Fig 2E, Table 3). Consistent classification accuracy above random was not achieved for all strata using the same modeling algorithms at a 2.5% quantile cutoff. For PCR and PLS modeling, equivalent quantiles of AUC were between 5.2–20.8% and 4.9–18.4% lower, respectively, compared to using signature metabolites. Thus, performance was inferior to using signature metabolites modeled using PLS or PCR (Table 3).

## Discussion

We have outlined an approach to identify individual metabolites strongly indicative of OA and a method of using them in classification modeling. Our results showed OA vs HV classification using metabolite levels was strongest in older males, resulting in a signature of 32 metabolites that individually had power to classify OA vs HV. Furthermore, we determined that modeling the signature using PCR with a single component resulted in the most consistent classification accuracy, as measured by mean AUC differences between bootstrap training and test sets. Finally, we concluded that using a signature-based list of metabolites and PCR/PLS modeling was more effective than aggregate modeling of all metabolites with the same methods, suggesting metabolite subsets are superior for detecting OA.

The statistical approach for this study (Fig 1) was chosen to reduce overfitting, allowing for greater confidence in the signatures we have identified and their potential classification power in independent datasets. With limited metabolomics research conducted on circulating serum or plasma from healthy and OA cohorts to date, validation has yet to be conducted on external cohorts. This is the first method in OA metabolomics research, to our knowledge, to focus on identifying cohort-specific signatures via demographic stratification. Independent cohort data will be necessary to conclusively determine if the modeling algorithm and identified signatures are universally predictive of OA.

Since metabolite signatures were strongest in the strata of males age > 50 years, metabolic processes may be more consistently modulated between OA and non-OA individuals in this stratum. Thus, in males, lysoPCs and PCs may be indicative of a response to OA pathophysiology or a direct contribution to symptoms/pathogenesis. The lack of this signal in females, given their higher general population incidence, may suggest varying etiology, a more biochemically heterogeneous disease population where metabolite signatures are less pronounced. However, in all of our metabolite signatures, confounding clinically-relevant variables were not controlled for in this study due to the lack of information from HV, a limitation of this study which may affect both signature elements and predictive outcomes. Controlling these confounding clinical variables using the method described herein could also increase both accuracy and precision of OA prediction.

We identified that compared to aggregates of metabolite types [7], select metabolite signatures are capable of classifying OA vs. HV in select strata. We also determined that this was as good as or better than aggregate levels alone, suggesting that select metabolites likely drive classification of individuals with OA. This is consistent with studies in other diseases where metabolite signatures were capable of classifying individuals with various pathologies including high-altitude pulmonary edema [13], multiple sclerosis [14] and pediatric tuberculosis [15].

Our study does however, have some limitations based on the clinical data that was available for use. For instance, we could not stratify based on metabolic disorders, including hyperlipidemia due to the lack of available clinical data, which could alter metabolite levels in the blood and be associated with hyperlipidemia as opposed to OA directly. Hyperlipidemia is a risk factor for OA [16] whereas metabolic syndromes are correlated with knee OA prevalence in select populations [17, 18]. Furthermore, although the incidence of diabetes mellitus is known in our cohort, we did not stratify or exclude individuals based on this factor, as we were unaware if this was well controlled by medication or lifestyle. Removing these individuals would also reduce the power for some of our strata. However, we found 4 metabolites that were significantly different in their levels between individuals with or without diabetes mellitus (DM) within the age > 50 years (3 metabolites) and males age > 50 years (1 metabolite) strata that overlapped with metabolites identified in our signatures from Table 2 (S1 Table). Removing these metabolites from our signatures and running PCR modeling of the remaining metabolites in the two affected strata resulted in minimal changes to the resulting AUCs (S2 Table). Curiously, there were more metabolites significantly different in the HV compared to the OA populations when comparing individuals with or without DM, suggesting that individuals with OA are more similar, even if they have DM (S1 Table). Overall, this suggests that the presence of DM is not a major confounding variable in our study.

Thus, we determined that individual lysoPC and PC analogues in plasma were collectively able to detect OA with some accuracy. Inclusion of other circulating biomarkers, like amino acids [6], cytokines [19] and microRNAs [20], into modeling algorithms may improve prediction.

## Supporting information

S1 Document R v3.1.1 coding used for dispersion tests, univariate AUC analysis and, multivariate regression AUC analysis. (DOCX)

S1 Table Metabolites significantly different between individuals with or without diabetes mellitus within healthy control volunteers or individuals with osteoarthritis stratified by age, body mass index (BMI; kg/m^2^) and sex. (DOCX)

S2 Table Quantiles of the principal component regression with logistic regression area under the multivariate receiver operator curve values of signatures from Table 2 with significantly altered metabolites identified in diabetic vs non-diabetic individuals (S1 Table) removed from the signatures. (DOCX)
