# Supplementary material for: A classification modeling approach for determining metabolite signatures in osteoarthritis
Source: PLoS One. 2018 Jun 29;13(6):e0199618. doi: 10.1371/journal.pone.0199618 (PMC6025859; doi:10.1371/journal.pone.0199618)
Supplement: S1 Document — (DOCX) [file pone.0199618.s001.docx]

#Packages used; R version 3.1.1

library(ROCR) #calculate AUC

library(MASS) #logistic regression functions

library(vegan) #dispersion tests

library(pls) #PLS and PCR regressions

###################################

#Estimating Metabolites Dispersions

###################################

#all.temp = dataset

#metab.list = list of metabolites

#all.temp$grp = list of groups as a factor to compare

met.incl <- decostand(all.temp[,metab.list], 1, method='norm') #normalize data

vare.dis <- vegdist(met.incl, method='eucl') #calculate normalized euclidean distances

disp.test <- betadisper(vare.dis, all.temp$grp) #estimate dispersion relative to groups

permutest(disp.test) #joint test

TukeyHSD(disp.test, pairwise=T) #pairwise comparisons

####################################################################################

################################

#Code to for a bootstrap iterate

################################

#all.set = dataset

#metab = metabolite name to select

#repeat N times for each metabolites to get an empirical distribution of AUC for each metabolite, repeat for each strata

#Randomly sample a training with replacement and define the test set (samples not included

boot.tr.set <- sample(1:dim(all.set)[1], dim(all.set)[1], replace=T)

boot.te.set <- setdiff(1:dim(all.set)[1], boot.tr.set)

tr.dat <- all.set[boot.tr.set,]; te.dat <- all.set[boot.te.set,]

#Fit the model on the training set and calculate AUC on the training set

fm.var <- as.formula(paste('Y ~ ', metab))

fit1 <- glm(fm.var, data=tr.dat, family=binomial('logit'))

lpred.fit <- predict(fit1, te.dat, type='response') #train the univariate model

auc.train <- get.perf(fit1$fitted.values, tr.dat$Y)$auc #predict training error

auc.test <- get.perf(lpred.fit, te.dat$Y)$auc #predict test error

#####################################

#Code for fitting multivariate models

#####################################

#fm.list = strata specific list of significant metabolites

#all.set = data for a particular strata

#repeat N times to get an empirical distribution of training and test set AUC for each scenario, repeat for each strata

#Calculate the performance of a training and test set

get.perf <- function(x1.train, x1.true) {

auc.tmp <- performance(prediction(x1.train, x1.true), 'auc')

return(as.numeric(auc.temp@y.values))

}

#Randomly sample a training with replacement and define the test set (samples not included

boot.tr.set <- sample(1:dim(all.set)[1], dim(all.set)[1], replace=T)

boot.te.set <- setdiff(1:dim(all.set)[1], boot.tr.set)

tr.dat <- all.set[boot.tr.set,]; te.dat <- all.set[boot.te.set,]

train.mat.iter <- tr.dat[,fm.list]; y.train.mat.iter <- tr.dat$Y

test.mat.iter <- te.dat[,fm.list]; y.test.mat.iter <- te.dat$Y

########################################################

#No Aggregation Logistic, PLS + Logistic, PCR + Logistic

########################################################

#Define the components and set of metabolites to use

tot.comps <- 1; pls.comps <- 1 #set the number of principal components to use

fm.var <- as.formula(paste('Y ~ ', paste(c(fm.list), collapse='+'))) #regression formula (outcome and short listed mets)

#### PLS + Logistic ####

pls.fit <- plsr(fm.var, data=tr.dat, ncomp=pls.comps) #train the PLS model on the raw data

pls.sc.te <- data.frame(predict(pls.fit, newdata=te.dat, type='scores')) #predict the scores for the test set

pls.sc.tr <- data.frame(pls.fit$scores[,1:pls.comps]) #pick top components

colnames(pls.sc.te) <- colnames(pls.sc.tr) <- colnames(pls.fit$scores)

pls.sc.tr$Y <- as.factor(y.train.mat.iter) #define scores dataframe to use for logistic training

pcr.fit <- pcr(fm.var, data=tr.dat, ncomp=tot.comps) #train the PCR model

pcr.sc.te <- data.frame(predict(pcr.fit, newdata=te.dat, type='scores')) #predict the scores for the test set

pcr.sc.tr <- data.frame(pcr.fit$scores[,1:tot.comps]) #pick top components

colnames(pcr.sc.te) <- colnames(pcr.sc.tr) <- colnames(pcr.fit$scores)

pcr.sc.tr$Y <- as.factor(y.train.mat.iter) #define scores dataframe to use for logistic training

#Differentiate via logistic (PLS)

pls.glm <- glm(Y ~ ., data=pls.sc.tr, family=binomial('logit')) #train classification using PLS scores

lpred.fit <- predict(pls.glm, pls.sc.te, type='response') #predict classification using test set scores

out.perf.train <- get.perf(pls.glm$fitted.values, y.train.mat.iter) #training error (PLS + Logistic)

out.perf.test <- get.perf(lpred.fit, y.test.mat.iter) #test error (PLS + Logistic)

#Differentiate via logistic (PCR)

pcr.glm <- glm(Y ~ ., data=pcr.sc.tr, family=binomial('logit')) #train classification using PCR scores

lpred.fit <- predict(pcr.glm, pcr.sc.te, type='response') #predict classification using test set scores

out.perf.train <- get.perf(pcr.glm$fitted.values, y.train.mat.iter) #traing error (PCR + Logistic)

out.perf.test <- get.perf(lpred.fit, y.test.mat.iter) #test error (PCR + Logistic)

#Logistic Regression only

fit1 <- glm(fm.var, data=tr.dat, family=binomial('logit')); summary(fit1) #train on all metabolites

lpred.fit <- predict(fit1, te.dat, type='response') #predict using test set

out.perf.train <- get.perf(fit1$fitted.values, tr.dat$Y) #training error

out.perf.test <- get.perf(lpred.fit, te.dat$Y) #test error

#####################################

#Summation of PC.aa, PC.ae and lysoPC

#####################################

#Define the metabolite summations

x.sum <- list()

x.sum[[1]] <- grep('PC.aa', fm.list)

x.sum[[2]] <- grep('PC.ae', fm.list)

x.sum[[3]] <- grep('lysoPC', fm.list)

x.add.tr <- lapply(x.sum, function(x){if (length(x) == 1){tr.dat[,fm.list][,x]} else if (length(x) > 1) {apply(tr.dat[,fm.list][,x], 1, sum)} else {0}}) #training set aggregates

x.add.te <- lapply(x.sum, function(x){if (length(x) == 1){te.dat[,fm.list][,x]} else if (length(x) > 1) {apply(te.dat[,fm.list][,x], 1, sum)} else {0}}) #test set aggregates

tr.sum.dat <- as.data.frame(array(0, dim=c(dim(tr.dat)[1], 3)))

te.sum.dat <- as.data.frame(array(0, dim=c(dim(te.dat)[1], 3)))

for (i in 1:3) {

tr.sum.dat[,i] <- x.add.tr[[i]]

te.sum.dat[,i] <- x.add.te[[i]]

}

colnames(tr.sum.dat) <- colnames(te.sum.dat) <- c('aa', 'ae', 'lyso')

#Set the formula to use in the regression (which aggregates to include)

fm.sum <- as.formula(paste('Y ~', paste(colnames(tr.sum.dat)[which(apply(tr.sum.dat, 2, sum) > 0)], collapse="+"), sep=''))

tr.sum.dat$Y <- tr.dat$Y

#Logistic Regression Only

fit1 <- glm(fm.sum, data=tr.sum.dat, family=binomial('logit')) #train logistic on summation of metabolites

lpred.fit <- predict(fit1, te.sum.dat, type='response') #predict on test set

out.perf.train <- get.perf(fit1$fitted.values, tr.dat$Y) #training set AUC

out.perf.test <- get.perf(lpred.fit, te.dat$Y) #test set AUC

#PLS + Logistic and PCR + Logistic - repeat using summation data

tot.comps <- 1; pls.comps <- 1 #total components for pcr and pls, respectively

pls.fit <- plsr(fm.sum, data=tr.sum.dat, ncomp=pls.comps) #train the PLS model

pls.sc.te <- data.frame(predict(pls.fit, newdata=te.sum.dat, type='scores')) #predict the scores

pls.sc.tr <- data.frame(pls.fit$scores[,1:pls.comps])

colnames(pls.sc.te) <- colnames(pls.sc.tr) <- colnames(pls.fit$scores)

pls.sc.tr$Y <- as.factor(y.train.mat.iter)

pcr.fit <- pcr(fm.sum, data=tr.sum.dat, ncomp=tot.comps)

pcr.sc.te <- data.frame(predict(pcr.fit, newdata=te.sum.dat, type='scores')) #predict the scores

pcr.sc.tr <- data.frame(pcr.fit$scores[,1:tot.comps])

colnames(pcr.sc.te) <- colnames(pcr.sc.tr) <- colnames(pcr.fit$scores)

pcr.sc.tr$Y <- as.factor(y.train.mat.iter)

#PLS + Logistic

pls.glm <- glm(Y ~ ., data=pls.sc.tr, family=binomial('logit'))

lpred.fit <- predict(pls.glm, pls.sc.te, type='response')

out.perf.train <- get.perf(pls.glm$fitted.values, y.train.mat.iter)

out.perf.test <- get.perf(lpred.fit, y.test.mat.iter)

#PCR + Logistic

pcr.glm <- glm(Y ~ ., data=pcr.sc.tr, family=binomial('logit'))

lpred.fit <- predict(pcr.glm, pcr.sc.te, type='response')

out.perf.train <- get.perf(pcr.glm$fitted.values, y.train.mat.iter)

out.perf.test <- get.perf(lpred.fit, y.test.mat.iter)
