# Supplementary material for: A classification modeling approach for determining metabolite signatures in osteoarthritis
Source: PLoS One. 2018 Jun 29;13(6):e0199618. doi: 10.1371/journal.pone.0199618 (PMC6025859; doi:10.1371/journal.pone.0199618)
Supplement: S1 Table — (DOCX) [file pone.0199618.s002.docx]

| **S1 Table.** Metabolites that are significantly different (p < 0.05 by students 2-tailed t-tests) between individuals with or without diabetes mellitus within healthy control volunteers (HV) or individuals with osteoarthritis (OA) stratified by age, body mass index (BMI; kg/m^2^) and sex. Yellow highlighted values correspond to matching metabolites found in signatures in Table 2. | | | | | | | | | | | |
| --- | --- | --- | --- | --- | --- | --- | --- | --- | --- | --- | --- |
| Group | Age > 50 | | Age >50 BMI > 30 | | Age > 50 BMI < = 30 | | Age > 50 Female | | Age > 50 Male | |  |
| Stratum | HV | OA | HV | OA | HV | OA | HV | OA | HV | OA |  |
| PC.aa.C28.1 | 0.008 |  |  |  | 0.002 |  | 0.031 |  |  |  |  |
| PC.aa.C30.0 | 0.026 |  |  |  | 0.026 |  | 0.019 |  |  |  |  |
| PC.aa.C32.0 | 0.015 |  |  |  |  |  | 0.014 |  |  |  |  |
| PC.aa.C32.3 |  |  |  |  |  |  | 0.006 |  |  |  |  |
| PC.aa.C34.2 |  |  |  |  |  |  | 0.020 |  |  |  |  |
| PC.aa.C34.3 | 0.028 |  |  |  |  |  | 0.001 |  |  |  |  |
| PC.aa.C36.0 |  |  | 0.031 |  |  |  |  |  |  |  |  |
| PC.aa.C36.1 | 0.044 |  |  |  |  |  |  |  |  |  |  |
| PC.aa.C36.2 | 0.017 |  |  |  |  |  | 0.012 |  |  |  |  |
| PC.aa.C36.3 |  |  |  |  |  |  | 0.038 |  |  |  |  |
| PC.aa.C36.5 |  |  | 0.044 |  |  |  |  |  |  |  |  |
| PC.aa.C36.6 | 0.021 |  | 0.007 |  |  |  | 0.015 |  |  |  |  |
| PC.aa.C38.0 |  |  | 0.009 |  |  |  | 0.013 |  |  |  |  |
| PC.aa.C38.3 | 0.002 |  | 0.029 |  | 0.044 |  | 0.039 |  | 0.020 |  |  |
| PC.aa.C38.4 |  |  |  |  |  |  |  |  | 0.049 |  |  |
| PC.aa.C38.6 | 0.014 |  | 0.010 |  |  |  | 0.014 |  |  |  |  |
| PC.aa.C40.3 | 0.039 |  | 0.010 |  |  |  | 0.013 |  |  |  |  |
| PC.aa.C40.5 | 0.004 |  |  |  | 0.014 |  |  |  | 0.003 |  |  |
| PC.aa.C40.6 | 0.001 |  | 0.006 |  | 0.030 |  | 0.013 | 0.043 | 0.022 |  |  |
| PC.aa.C42.0 |  |  |  |  |  |  |  |  |  | 0.042 |  |
| PC.aa.C42.2 |  |  |  |  |  |  | 0.041 |  |  |  |  |
| PC.ae.C32.1 |  |  | 0.032 |  |  |  |  |  |  |  |  |
| PC.ae.C32.2 |  |  |  |  |  |  | 0.015 |  |  |  |  |
| PC.ae.C34.0 |  |  |  |  |  |  | 0.020 |  |  |  |  |
| PC.ae.C34.2 |  |  |  |  |  |  | 0.020 |  |  |  |  |
| PC.ae.C34.3 |  |  |  |  |  |  | 0.009 |  |  |  |  |
| PC.ae.C36.3 |  |  |  |  |  |  | 0.030 |  |  |  |  |
| PC.ae.C36.5 |  |  |  |  |  |  | 0.030 |  |  |  |  |
| PC.ae.C38.0 |  |  | 0.021 |  |  |  | 0.016 |  |  |  |  |
| PC.ae.C38.1 |  |  |  | 0.049 |  |  |  |  |  |  |  |
| PC.ae.C38.6 |  |  |  |  |  |  | 0.044 |  |  |  |  |
| PC.ae.C40.1 |  |  | 0.030 |  |  |  |  |  |  |  |  |
| PC.ae.C42.0 |  |  |  |  |  |  |  | 0.016 |  |  |  |
| PC.ae.C42.1 | 0.003 |  | 0.007 |  |  |  | 0.030 |  | 0.018 |  |  |
| PC.ae.C42.3 |  |  |  |  |  |  | 0.044 |  |  |  |  |
| PC.ae.C44.3 |  |  |  |  |  |  |  |  |  | 0.048 |  |
| PC.ae.C44.5 |  |  |  |  |  |  |  |  |  | 0.049 |  |
| Sample Size (N) | 102 | 148 | 42 | 89 | 60 | 59 | 52 | 76 | 50 | 72 |  |

Age (in years), (lysoPC), diacyl phosphatidylcholine (PCaa), acyl-alkyl PC (PCae).
