# Supplementary material for: A classification modeling approach for determining metabolite signatures in osteoarthritis
Source: PLoS One. 2018 Jun 29;13(6):e0199618. doi: 10.1371/journal.pone.0199618 (PMC6025859; doi:10.1371/journal.pone.0199618)
Supplement: S2 Table — (DOCX) [file pone.0199618.s003.docx]

| **S2 Table.** Quantiles of the principal component regression with logistic regression (PCR) area under the multivariate receiver operator curve values of signatures from Table 2 with significantly altered metabolites identified in diabetic vs non-diabetic individuals (yellow highlighted in S1 Table) removed from the signatures. | | | | | | |
| --- | --- | --- | --- | --- | --- | --- |
| Stratum | All age > 50 | | | Males age > 50 | | |
| Model | Training | Test | Difference | Training | Test | Difference |
| PCR 2.5% | 0.622 | 0.596 | -0.026 | 0.656 | 0.623 | -0.032 |
| PCR 50% | 0.685 | 0.683 | -0.002 | 0.750 | 0.755 | 0.005 |
| PCR 97.5 | 0.751 | 0.761 | 0.010 | 0.829 | 0.866 | 0.037 |

Age (in years).
